# Current Status of CRISPR/Cas9 Application in Clinical Cancer Research: Opportunities and Challenges

**DOI:** 10.3390/cancers14040947

**Published:** 2022-02-14

**Authors:** Saeed Rafii, Emad Tashkandi, Nedal Bukhari, Humaid O. Al-Shamsi

**Affiliations:** 1 Department of Oncology, Saudi German Hospital, Dubai P.O. Box 391093, United Arab Emirates; oncologydoc3.dxb@saudigerman.com; 2 Emirates Oncology Society, Dubai P.O. Box 6600, United Arab Emirates; 3 Oncology Center, King Abdullah Medical City, Makkah P.O. Box 24246, Saudi Arabia; tashkandi.e@kamc.med.sa; 4 Department of Medicine, College of Medicine, Umm Al Qura University, Makkah P.O. Box 24382, Saudi Arabia; 5 Department of Medical Oncology, King Fahad Specialist Hospital, Dammam P.O. Box 31444, Saudi Arabia; 6 Department of Internal Medicine, Imam Abdulrahman Bin Faisal University, Dammam P.O. Box 34212, Saudi Arabia; nedal.bukhari36@gmail.com; 7 Department of Oncology, Burjeel Cancer Institute, Burjeel Medical City, Abu Dhabi P.O. Box 92510, United Arab Emirates; 8 Innovation and Research Center, Burjeel Cancer Institute, Burjeel Medical City, Abu Dhabi P.O. Box 92510, United Arab Emirates; 9 College of Medicine, University of Sharjah, Sharjah P.O. Box 27272, United Arab Emirates

**Keywords:** CRISPR-Cas9, cancer therapy, CAR-T

## Abstract

**Simple Summary:**

It is widely believed that cancer is developed due to changes in the genetic codes of our DNA, leading to abnormal growth of cells. In the past few years scientists have discovered a system which is used as an immune mechanism by bacteria in order to cleave the invading viruses, called CRISPR/Cas9. Exploiting this system in humans will allow scientists to attempt to edit genetic errors that lead to cancer. This scientific breakthrough has a lot of potential for treating a variety of diseases, including cancer. It has already been successfully used in treatment of some types of blood cancer. In this article, we discuss the opportunities and limitations of CRISPR/Cas9 in treatment of solid cancer.

**Abstract:**

Cancer is considered by not only multiple genetic but also epigenetic amendments that drive malignant cell propagation and consult chemo-resistance. The ability to correct or ablate such mutations holds enormous promise for battling cancer. Recently, because of its great efficiency and feasibility, the CRISPR-Cas9 advanced genome editing technique has been extensively considered for therapeutic investigations of cancers. Several studies have used the CRISPR-Cas9 technique for editing cancer cell genomic DNA in cells and animal cancer models and have shown therapeutic potential in intensifying anti-cancer protocols. Moreover, CRISPR-Cas9 may be used to correct oncogenic mutations, discover anticancer drugs, and engineer immune cells and oncolytic viruses for immunotherapeutic treatment of cancer. We herein discuss the challenges and opportunities for translating therapeutic methods with CRISPR-Cas9 for clinical use and suggest potential directions of the CRISPR-Cas9 system for future cancer therapy.

## 1. Introduction

### What Is CRISPR/Cas9?

**Figure 1:**
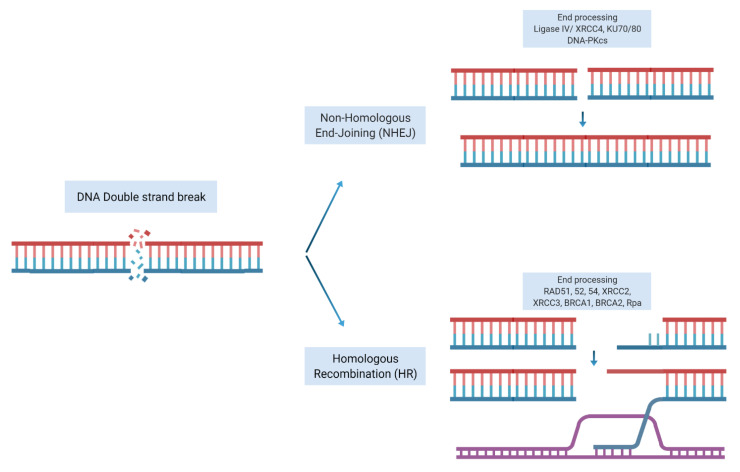
Repair of double strand breaks. Non-homologous end joining repair: a double-strand break is rejoined end-to-end. Homologous recombination repair: a double-strand break is repaired with the help of homologous undamaged DNA (shown in orange). Strand invasion allows re-synthesis on a complementary sequence, followed by a resolution of the strands and rejoining. Adapted from [1]. Figure created with BioRender.com accessed on 11 December 2021).

Our DNA is constantly being damaged by internal and external sources. It is widely accepted that genomic instability and accumulation of driver aberrations lead to clonal cancer evolution. Mammalian cells have developed systems to repair and maintain the integrity of their genome through employing various DNA damage response (DDR) mechanisms. DNA double strand breaks (DSB) are considered one of the most lethal insults to cells. Following induction of DSB, the DNA repair machinery is activated to repair the DSB. There are two main pathways for repairing a DSBs, non-homologous end joining (NHEJ) or homologous recombination repair (HRR). The NHEJ repair system simply processes the broken DNA ends, joins the two ends of DSB and ligates the two damaged ends together. Although an efficient repair mechanism, it gives rise to mutations, deletions, or insertions (Indels). In contrast, HRR is an accurate repair mechanism that uses the homologous sister chromatid as a template to precisely repair the DSB. This system requires a complex and coordinated machinery of proteins and enzymes to first resect the damaged ends of DNA, identify and localize the homologous sister chromatid at the site of the damage and facilitate the invasion of 3′ single-stranded tails of the sister homologous chromatid into the damaged DNA to act as a template to restore the damage to its original sequence using DNA polymerases (Figure 1).

Gene editing has a long history and technologies such as zinc finger nucleases (ZFNs) and transcription activator-like effector nucleases (TALENs) have previously been described [2]. Such platforms use a zinc finger protein or a transcription activator-like effector (TALE) in order to identify a target DNA sequence and perform endonuclease activity to induce a DSB. However, both of these platforms have limitations that hinder their routine usage in basic or clinical research. For example, ZFN is associated with a high rate of off-target cleavage and the mode of the delivery of TALEN to cells is limited due to its long DNA recognition sequence [3,4,5].

**Figure 2:**
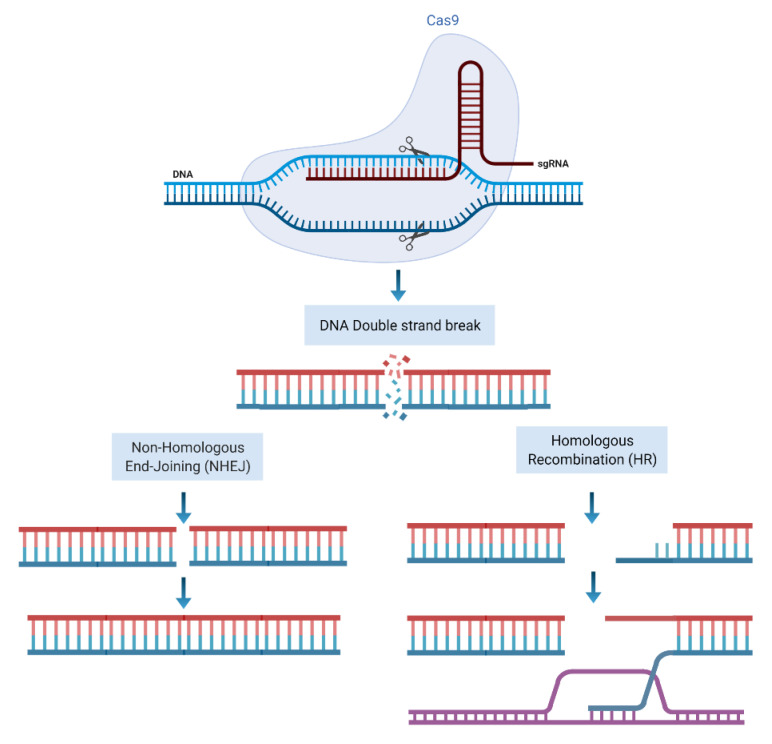
CRISPR/Cas9 mode of action. sgRNA identifies the target sequence and CAS9 endonuclease activity makes a double stranded DNA cleavage downstream of PAM. DSBs then can be repaired through NHEJ or HR repair machineries. Figure created with BioRender.com accessed on 11 December 2021).

Clustered regularly interspaced short palindromic repeat (CRISPR)-associated protein-9 (Cas9) is a gene editing tool that comprised of a single guided RNA (sgRNA) and a DNA endonuclease Cas9 protein. The sgRNA identifies the matched complimentary sequence of the genome and guides the Cas9 to the site of target DNA [6]. Following pairing of sgRNA with the target sequence Cas9 nuclease domains makes site specific double-stranded breaks close to a section of sgRNA called proto-spacer adjacent motif (PAM) located at the 3′ end of sgRNA [7]. Following induction of DSB, cell repair machinery is activated to repair and restore the genome through NHEJ or HRR pathways, as explained below (Figure 2).

Multiple sgRNA sequences can, in principle, allow gene editing at multiple locations [8]. This ability provides the potential for scientists to speculate as to the possibility of simultaneous multiple deletions and insertions of base pairs, which has huge potential to enable treatment for some inherited disorders that have previously been deemed incurable. Can we exploit such a flexible and high-fidelity repair mechanism to correct mutational errors and treat cancer? Gene editing and, in particular, CRISPR/Cas9 technology has turned into an area of interest for many scientists and clinicians alike in order to study its potential in cancer research and treatment.

## 2. How Can CRISPR Technology Be Used in Cancer Research?

CRISPR/Cas9 technology offers significant potential in laboratory cancer research, including the ability to generate better disease models for cancer, drug targets and discovery and study of treatment resistance. It is a flexible, relatively simple-to-use technology with a high degree of efficacy which is affordable and easy to use in laboratory research [9].

CRISPR/Cas9 has been successfully used to inactivate several tumor suppressor genes in order to create cancer disease models. For example, CRISPR-induced deletion of P53, Nf1, Pten and Ptch1 in a mouse brain resulted in forming medulloblastoma and glioblastoma [10]. As discussed above, cancer cells accumulate a series of mutations and genomic aberrations over time. CRISPR/Cas9 gives us the opportunity to create cancer models with different mutations to identify early events that lead to mutagenesis and genome instability. For example, CRISPR/Cas9 has been used to identify truncating events in the APC gene as an early event in developing colorectal cancer [11,12]. Another example is knockout of HER2 exon 12, which subsequently produced a truncated HER-2 protein with knockout HER-2 protein expression, suggesting that a partial oncogene knockout may be enough in order to induce a therapeutic impact [13]. CRISPR/Cas9 technology is also being used in order to restore tumor suppressor gene functions, for example demethylation of PTEN and BRCA1 genes, which leads to better response to chemotherapy [14,15], or correcting mutations that lead to inactivation of tumor suppressor genes [16]. Loss of PTEN expression is frequently seen in many cancers. Increasing PTEN expression via CRISPR in melanoma and triple negative breast cancer (TNBC) cell lines has been associated with reducing downstream oncogenic MAP kinase activity [14].

Drug resistance is a significant issue in oncology. CRISPR/Cas9 technology has provided an opportunity to study the mechanism of resistance to anti-cancer drugs by correcting activating mutations that confer resistance to certain therapies. Examples of using CRISPR/Cas9 include models of EGFR exon 20 mutations to study the efficacy of the EGFR tyrosine kinase inhibitor (TKI) osimertinib [17], or ablation of Aurora-B that re-sensitizes resistant lung cancer cell lines to cisplatin [18]. CRISPR/Cas9 technology has also been instrumental in identifying multiple gene mutations resulting in resistance to treatment in breast cancer such as ESR1 mutation and resistance to fulvestrant or new mechanisms of resistance to PARP inhibitors [19,20,21].

Enhanced toxicities from combining anticancer drugs are a major factor in anticancer drug development. For instance, despite the synergistic effect of platinum and PARP inhibition, such concurrent combination strategies have largely failed, mainly due to high grade myelosuppressive toxicities. It has been demonstrated that CRISPR-induced inhibition of PARP1 synergizes cisplatin cytotoxicity in ovarian cancer [22], which may lead to a potential concurrent platinum and PARP inhibition, instead of using small molecule inhibitors. Several kinase proteins such as PIK3CA, BRAF, KRAS may be subject to knockout by CRISPR/ Cas9 where targeting activating kinase domain of their proteins may not be feasible or could be too toxic for clinical use.

## 3. What Is the Current Status of Using CRISPR/Cas9 in Cancer Treatment?

In the last decade, the field of oncology has witnessed a revolution in the way we treat patients with cancer. Immunotherapy remains at the forefront of this therapeutic evolution. Immune checkpoint blockade, CAR-T cells, and adaptive T cell therapy (ACT) are among the treatments that have successfully entered the clinical setting.

### 3.1. CRISPR/Cas9 and CAR-T Cell Therapies

Chimeric antigen receptor (CAR) T cells (CAR-T) are harvested T cells that have been genetically engineered in order to express receptor to recognize neoantigens that are expressed on the cancer cells surface [23]. Such tumor-targeting T cells have attracted a lot of interest. CARs consist of an extracellular domain that identifies target surface antigens, and an intracellular T cell signaling domain that triggers CAR-T cell activation [24]. A new generation of CAR-T cells has built-in co-stimulatory intracellular domains that allow expansion, proliferation and survival of T cells [25,26]. Most CARs identify surface antigens independent of the major histocompatibility complex (MHC). Many factors determine the performance of CARs, including fine tuning of the intracellular and extracellular domains as well as the non-functional connecting transmembrane domain and hinges [27], and location on target cell surface antigen [28].

While CAR-T cells have been very successful in the treatment of hematological malignancies, particularly B cell malignancies [29,30], their applications in solid malignancies have largely been unsuccessful so far. Multiple reasons are responsible for the lack of effectiveness of CAR-T cell therapy in solid cancers. One of the most important factors is the lack of enough surface antigens in solid cancers compared with hematological malignancies. Most neoantigens in solid tumors are intracellular and therefore not suitable targets for CAR-T cell therapy [31]. Although multiple efforts are underway to increase the affinity and specificity of target antibody engagement [32,33], tumor heterogeneity in solid cancer leads to heterogenous antigen expression and eventually to immune escape [34]. Some cancers lack the tumor mutational burden and MHC expression, which hinders the application of conventional CARs. Very recently, a synthetic peptide-centric CARs (PC-CARs) approach has been proposed to target some tumor specific oncoproteins [35].

Another obstacle is T cell exhaustion, which is the result of chronic exposure to antigens [36]. Several strategies have been employed to increase T cell fitness, such as provision of additional co-stimulatory signals [37], transgenic expression of cytokines [38], and silencing of inhibitory molecules [39]. Another major hurdle in developing CAR-T cell therapy in solid tumors is T cell exclusion. Migration of CAR-T into solid tissue is influenced by multiple factors such as dense tumor stroma, chemokines, tumor vasculature and tumor infiltrating immune cells [40,41]. In addition to all these, the tumor microenvironment and its suppressive immunomodulatory components, such as cancer-associated fibroblasts and cytokines, result in depletion of T cells and their fitness [42,43].

Such limitations, as well as the high rate of toxicities, including cytokine release syndrome (CRS) and the risk of graft versus host disease (GVHD), have hindered the development of CAR-T cell therapies in treatment of solid cancers [43]. The CRISPR/Cas9 platform and its multiplexing ability has given us the opportunity to overcome some challenges of using CAR-T cell therapy, including silencing of HLA-I and TCR of allogenic T cells in order to prevent graft rejection associated with allogenic T cells, paving the way to using off-the-shelf CAR-T cells [44]. It can also be used to specifically modify certain cytokine genes in order to enhance cytokine production and immune cell response, therefore avoiding T cell exhaustion and autoimmune response which is associated with other methods of cytokine enhancement such as viral transduction [39]. Additionally, the CRISPR/Cas9 platform can be used to knockout checkpoint inhibitor genes such as those coding for PD-1 and CTLA-4 for longer-lasting T cell engagement [45].

### 3.2. CRISPR/Cas9 and Adaptive T Cell Therapies

Adaptive cell therapy (ACT) has the potential to overcome the reliance on tumor infiltrating lymphocytes (TILs) that are not always found in all tumors. The T cell receptor (TCR) complex is at the core of these treatment modalities and is responsible for “foreign antigens”. In ACT, patients’ T cells are engineered to express particular TCRs that are able to detect antigens expressed by tumor cells, and direct T cell killing of the tumor cells. Such transgenic TCRs specifically engineered to recognize NY-ESO1 antigens in certain tumors provide an opportunity for targeted killing of tumor cells presenting those immunogenic antigens. A potential problem with ACT is the mispairing of α and β chains of the therapeutic and endogenous TCR complex resulting in reduced efficacy for engaging TCR with the target antigen. Additionally, the negative regulatory effect of PD-1 expression on T cells reduces the antigen response and therefore the efficacy of T-cell-directed tumor killing. Preclinical mouse model studies indicate that a combination of PD-1 blockade and transgenic TCR T cells against NY-ESO1 antigen presenting tumor cells is associated with improved efficacy [46]. This has led to the first phase I clinical trial, which tested the safety and feasibility of multiplex CRISPR/Cas9 to delete gene loci encoding for TCR α and β chains in a synthetic transgenic TCR in order to reduce their mispairing effects, and also to make deletion in PDCD1 gene locus that encodes for PD-1. Using this triple edited TCR, the investigators treated three patients with refractory cancer including two patients with treatment refractory multiple myeloma and one patient with advanced metastatic liposarcoma [47]. All patients received lymphodepleting chemotherapy with cyclophosphamide and fludarabine 3 to 5 days prior to infusion of CRISPR/Cas9 engineered T cells. The investigators reported treatment related adverse events to be mainly due to myelosuppressing chemotherapy, including grade 3 and 4 hematological toxicities. Non-hematological toxicities were mainly low grade and consisted of pain, paresthesia, headache, cough, and GI disturbances. Use of engineered transgenic TCR was associated with no cytokine release syndrome. The investigators reported sustained persistence of engineered T cells in the blood of all three patients up to 9 months after infusion with the decay half-life of up to 293 days. Additionally, the investigators demonstrated successful trafficking of engineered T cells in the bone marrow and tumor biopsy of patients at a level that was comparable to the blood level. Regarding the specificity of on- and off-target cleavage, although off-target nuclease activities were observed resulting in mutations and translocations, they were not associated with any cell growth advantage up to 300 days of expansion in vivo [47].

**Figure 3:**
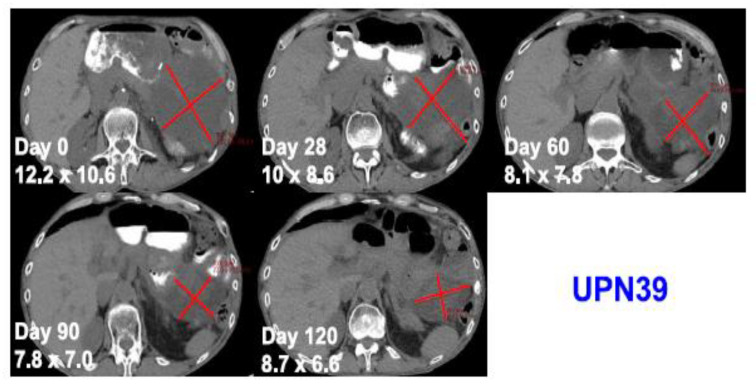
Computed tomography scans of a patient with metastatic treatment refractory liposarcoma showing tumor regression after administration of an autologous NYCE T cell. Adapted from [47] with permission from the authors.

One patient with advanced and treatment refractory metastatic liposarcoma showed mixed response to treatment with a large abdominal lesion showed 50% reduction in the size of the disease with the duration of response lasting for 4 months, while other metastatic lesions progressed (Figure 3). The best response was stable disease and eventually all three patients developed progressive disease. This study is the first study to provide the safety and feasibility of CRISPR/Cas9 gene editing for the use in patients with cancer, including a patient with solid malignancy.

The second clinical trial using CRISPR/Cas9 PD-L1 edited T cells was conducted in advanced treatment of refractory non-small-cell lung cancer. Of twenty-two patients enrolled in the trial, twelve patients received the edited T cell infusion. The investigators reported that the treatment was safe and that all treatments related to adverse events were low grade. The editing efficiency in the infused cells was low, which could be due to the delivery method rather than the gene editing issue. This resulted in short term persistence of the edited T cells in patients. The off-target mutation frequency was low (0.05%, range 0–0.25%), evaluated by next generation sequencing at 18 potential off-target loci. None of the patients showed objective response to treatment, although one patient with higher PD-1 editing efficiency showed stable disease for 76 weeks, indicating that better editing efficiency may lead to greater antitumor activity [48].

These two studies have addressed the safety concerns associated with the use of CRISPR/Cas9 technology to some extent, as well as providing the proof of principle for the feasibility of CRISPR/Cas9 as a therapeutic option for solid and hematological malignancies. Multiple clinical trials are currently underway to assess safety and antitumor activity of CRISPR/Cas9 edited T cells in multiple cancers (Table 1).

## 4. Challenges of Using CRISPR Technology in Cancer Therapy

### 4.1. Methods of Delivery of CRISPR-Cas9 into Cells

While CRISPR/Cas9-directed ex vivo gene modification has been very promising, in vivo delivery of CRISPR/Cas9 into solid malignancies is possibly the biggest challenge ahead. A few delivery methods have recently been used, such as viral vectors like adeno-associated virus and lentivirus, or non-viral delivery methods like lipid- or polymer-based nanoparticles [49,50,51]. Intratumoral injection of CRISPR/Cas9 has also been studied for localized treatment of TNBC in mouse models, demonstrating target genome efficacy and reduction in the tumor volume [13]. Each of these delivery systems has its own limitations and challenges [52,53]. A general challenge with any delivery method is to achieve a high concentration of CRISPR/Cas9 in the target tissue that can reach all target cells. At the moment, it seems unlikely that any delivery system can achieve a hundred percent target cell delivery, which in turn may lead to treatment failure. Therefore, developing a delivery system that allows direct infusion of CRISPR/Cas9 into humans and a safe and efficient high concentration intratumoral delivery will certainly be a major step up in applying CRISPR/Cas9 for therapeutic use in solid oncology.

### 4.2. Target Specification

Another major challenge with CRISPR/Cas9 technology is off-target gene editing. Off-target DNA strand cleavages leading to off-target mutations and larger chromosomal rearrangements, such as inversions and translocations, are observed in all gene editing platforms [44,54]. In CRISPR/Cas9, the off-target mutations are commonly due to PAM and sgRNA mismatches. To reduce the chance of sgRNA mismatch, sophisticated software is used to predict off-target cleavage sites [55]. Additionally, as well as PCR and next-generation sequencing, newer and more sensitive methods such as UDiTaS^TM^ are in development to monitor the off-target mutations more accurately [56]. One observation in cells that have competent *P53* is that CRISPR/Cas9 gene modification may not be optimal due to the tumor suppressor effect of *P53* [57]. Two studies suggest that the efficiency of CRISPR/Cas9 gene modification is more enhanced in *TP53* mutated cells [58,59]. This has raised some concerns that disrupting *TP53* may lead to mutations and DNA rearrangements in proto-oncogenes which may subsequently increase the chance of malignancy. These concerns regarding the safety of the CRISPR/Cas9 system are the subject of extensive research to increase the site specificity and reducing off target nuclease activities. Among the strategies currently being researched are more advanced editing techniques such as base and prime editors that reduce the chance of off-target mutations [60]. Base editing techniques allow synthetic mutations with single nucleotide changes, essentially allowing a targeted base change in the DNA [61]. One advantage of base editing technology is that it does not rely on DSBs and therefore eliminates the chance of chromosomal rearrangements. Another flexible platform is prime editing, which uses a Cas9 variant fused to a reverse transcriptase and a guided RNA. It benefits from a guided sequence at the 5′ end and a primer binding site at the 3′ end with an RNA template that edits and replaces the desired sequence. This versatile platform reduces the chance of relying on the DSB and reduces off-target chromosomal rearrangements [62].

## 5. Conclusions

The CRISPR/Cas9 gene editing platform holds great research and clinical promise in cancer therapeutics. This simple and versatile system has the ability to help us understand the mechanisms of cancer predisposition and mechanism of metastasis, as well as predicting response to treatment and drug resistance. In the clinical context, it is set to revolutionize the use of CAR-T and adaptive cell therapies by overcoming some hurdles such as GVHD and T cell exhaustion. There are considerable challenges ahead of developing CRISPR/Cas9 technology as a routine treatment of solid cancer. Manufacturing timeline, high cost of manufacturing, off-tumor effects, CAR-T delivery, trafficking and tumor infiltration, and associated safety and toxicities are a few examples of the major challenges ahead. Inherent clonal selection and expansion of cancer which lies at the center of therapeutic failure for the majority of anticancer therapies, including chemotherapy and targeted therapy, is another major obstacle. Even with effective and safe CRISPR/Cas9 targeting, tumor heterogeneity and presence of sub clones which harbor different driver mutations may lead to the emergence of resistant clones. Identification of multiple sub clones before treatment and multiplex CRISPR/Cas9 sgRNA may be one way to address this problem to some extent. Another significant challenge is how CRISPR/Cas9 might be useful in treating cancers caused by structural events or copy number aberrations. Nonetheless, more clinical trials are underway to address these points which we expect to set the gene editing technology as an integral part of cancer treatment in the future.

## Figures and Tables

**Table 1:** Key ongoing or recent clinical trials using CRISPR technology in solid and hematologic malignancies.

Agent	Clinical Trial *	Sponsor	*n* ^†^, Population, Age	Primary Outcomes Measures
Neoantigen-specific TIL edited with CRISPR/Cas-9 to inhibit intracellular immune checkpoint CISH	NCT04426669 Phase 1/2, non-randomized, sequential assignment Recruiting	Intima Bioscience, Inc., New York, NY, USA	*n* = 20 Metastatic gastrointestinal epithelial cancer with progressive disease following at least one first-line standard therapy 18–70 yr	MTD Tumor diameter AE
CD19-specific CAR-T cells edited with CRISPR guide RNA to disrupt expression of endogenous HPK1 (XYF19 CAR-T cell)	NCT04037566 Phase 1, non-randomized, single group assignment Recruiting	Xijing Hospital, China	*n* = 40 Relapsed/refractory CD19+ B-cell leukemia or lymphoma 18–55 yr	AE MTD/DLT
NY-ESO-1 redirected autologous T cells and edited with CRISPR guide RNA to disrupt expression of TCR and PD-1 (NYCE T Cells)	NCT03399448 Phase 1, non-randomized, parallel assignment Terminated	University of Pennsylvania, USA	*n* = 3 Relapsed/refractory multiple myeloma, melanoma, synovial sarcoma, or myxoid/round cell liposarcoma ≥18 yr	AE Manufacturing feasibility
CD34+ hematopoietic stem/progenitor cells with CRISPR/Cas9 disruption of CCR5	NCT03164135 Phase 1, non-randomized, single group assignment Unknown	Peking University Affiliated Hospital to Academy of Military Medical Sciences, China	*n* = 5 HIV-infected hematologic malignancies 18–60 yr	Persistence of CCR5 gene disruption in engrafted cells
Mesothelin-directed CAR-T cells with CRISPR/Cas9 mediated PD-1 and TCR knock out	NCT03545815 Phase 1, non-randomized, single group assignment Recruiting	Chinese PLA General Hospital, China	*n* = 10 Mesothelin positive solid tumors with failure of at least one prior standard of care chemotherapy for advanced stage disease 18–70 yr	AE Disease control rate
CD19-specific CAR-T cells with chRDNA integrated CD19-CAR at TRAC and PD-1 knock out (CB-010)	NCT04637763 (CB010A) Phase 1, non-randomized, sequential assignment Recruiting	Caribou Biosciences, Inc., Berkeley, CA, USA	*n* = 50 Relapsed/refractory NHL after prior standard of care ≥18 yr	DLT Objective response rate
CD19-specific CAR-T cells with CRISPR/Cas9 disruption of B2M, CIITA, and TRAC (PACE CART19)	NCT05037669 Phase 1, non-randomized, sequential assignment Not yet recruiting	University of Pennsylvania, USA	*n* = 36 Relapsed/refractory ALL, CLL, NHL ≥18 yr	Recommended expansion dose
TALEN and CRISP/Cas9 disrupted HPV 16/18 E6/E7	NCT03057912 Phase 1, non-randomized, parallel assignment Unknown	First Affiliated Hospital, Sun Yat-Sen University, China	*n* = 60 Women with HPV16 or HPV18 infection at risk of HPV-related cervical intraepithelial neoplasia 18–50 yr	AE
BCMA-directed T-cell immunotherapy modified ex vivo using CRISPR/Cas9 (CTX120)	NCT04244656 Phase 1, non-randomized, sequential assignment Recruiting	CRISPR Therapeutics AG, Switzerland/USA	*n* = 80 Relapse/refractory multiple myeloma ≥18 yr	AE Objective response rate
CD70-directed T-cell immunotherapy modified ex vivo using CRISPR/Cas9 (CTX130)	NCT04438083 (COBALT-RCC) Phase 1, non-randomized, sequential assignment Recruiting	CRISPR Therapeutics AG, Switzerland/USA	*n* = 107 Unresectable or metastatic renal cell carcinoma that has exploited standard of care treatment ≥18 yr	AE Objective response rate
CD70-directed T-cell immunotherapy comprised of allogeneic T cells genetically modified ex vivo using CRISPR-Cas9 gene editing components	NCT04502446 (COBALT-LYM) Phase 1, non-randomized, sequential assignment Recruiting	CRISPR Therapeutics AG, Switzerland/USA	*n* = 45 T cell malignancy or DLBCL ≥18 yr	AE Objective response rate
CD19-specific CAR-T cells with CRISPR/Cas9 edited CD52 and TRAC (PBLTT52CAR19)	NCT04557436 Phase 1, non-randomized, single group assignment Recruiting	Great Ormond Street Hospital for Children NHS Foundation Trust, UK	*n* = 10 Relapsed/refractory CD19+ B-cell ALL 6 months-18 yr	Remission
Mesothelin-directed CAR-T cells with CRISPR/Cas9 mediated PD-1 knock out	NCT03747965 Phase 1, non-randomized, single group assignment Unknown	Chinese PLA General Hospital, China	*n* = 10 Mesothelin positive solid tumors (especially pancreatic cancer, cholangiocarcinoma, ovarian cancer) with failure of at least one prior standard of care chemotherapy for advanced stage disease 18–70 yr	AE Disease control rate
CD19 and CD20 or CD22-specific CRISPR/Cas9 edited CAR-T cells	NCT03747965 Phase 1/2, non-randomized, single group assignment Recruiting	Chinese PLA General Hospital, China	*n* = 80 Relapsed/refractory B-cell leukemia or lymphoma 12–70 yr	AE MTD CAR copies
CD19-directed T-cell immunotherapy modified ex vivo using CRISPR/Cas9 (CTX110)	NCT04035434 (CARBON) Phase 1, non-randomized, sequential assignment Recruiting	CRISPR Therapeutics AG, Switzerland/USA	*n* = 143 Relapsed/refractory B-cell ALL or NHL ≥18 yr	AE Objective response rate
CD19-specific CRISPR/Cas9 edited CAR-T cells(UCART019)	NCT03166878 Phase 1/2, non-randomized, single group assignment Recruiting	Chinese PLA General Hospital, China	*n* = 80 Relapsed/refractory CD19+ B-cell leukemia or lymphoma 12–75 yr	AE DLT UCAR019 copies
T cells with CRISPR/Cas9 PD-1 knock out (PD-1 Knockout EBV-CTL)	NCT03044743 Phase 1/2, non-randomized, single group assignment Recruiting	Yan Yang, China	*n* = 20 EBV positive stage IV gastric carcinoma, nasopharyngeal carcinoma and lymphoma 18–75 yr	AE
T cells with CRISPR/Cas9 PD-1 knock out	NCT02793856 Phase 1, non-randomized, parallel assignment Completed	Sichuan University, China	*n* = 12 Stage IV non-small-cell lung cancer 18–70 yr	AE
T cells with CRISPR/Cas9 PD-1 knock out combined with transcatheter arterial chemoembolization	NCT04417764 Phase 1, non-randomized, single group assignment Recruiting	Central South University, China	*n* = 10 Unresectable hepatocellular carcinoma 18–70 yr	AE

* Status per clinicaltrials.gov on 19 September 2021. ^†^ Actual or estimated, per clinicaltrials.gov on 19 September 2021. CRISPR, clustered regularly interspaced short palindromic repeats; Cas9, CRISPR-associated protein 9; TIL, tumor infiltrating lymphocytes; CISH, cytokine-induced SH2 protein; yr, years; MTD, maximum tolerated dose; DLT, dose-limiting toxicity; AE, adverse events; CAR-T, chimeric antigen receptor T cells; HPK1, hematopoietic progenitor kinase 1; TCR, T cell receptor; PD-1, programmed cell death protein-1; NY-ESO-1, New York esophageal squamous cell carcinoma-1; CCR5, C-C chemokine receptor type 5; HIV, human immunodeficiency virus; TRAC, T cell receptor alpha constant; GvHD, graft versus host disease; chRDNA, CRISPR hybrid RNA-DNA; B2M, beta-2 microglobulin; CIITA, class II major histocompatibility complex transactivator; ALL, acute lymphoblastic leukemia; CLL, chronic lymphocytic leukemia; NHL, non-Hodgkin’s lymphoma; HPV, human papillomavirus; TALEN, transcription activator-like effector nucleases; BCMA, B-cell maturation antigen; DLBCL, diffuse large B-cell lymphoma; EBV, Epstein–Barr virus.

## Abbreviations

CRISPR: Clustered Regularly Interspaced Short Palindromic Repeat Associated Protein-9 (Cas9); DDR: DNA Damage Response; DSB: DNA Double Strand Breaks; NHEJ: Non-Homologous End Joining; HRR: Homologous Recombination Repair; ZFN: Zinc Finger Nucleases; TALENs: Transcription Activator- Like Effector Nucleases; PAM: Proto-Spacer Adjacent Motif; TNBC: Triple Negative Breast Cancer; TKI: Tyrosine Kinase Inhibitor; PARP: Poly (ADP-ribose) polymerase; ACT: Adaptive T Cell Therapy; CAR-T: Chimeric Antigen Receptor T-Cells; MHC: Major Histocompatibility Complex; CRS: Cytokine Release Syndrome; GVHD: Graft Versus Host Disease; TIL: Tumor Infiltrating Lymphocytes; TCR: T Cell Receptor.
